# Motion Capture Sensor-Based Emotion Recognition Using a Bi-Modular Sequential Neural Network

**DOI:** 10.3390/s22010403

**Published:** 2022-01-05

**Authors:** Yajurv Bhatia, ASM Hossain Bari, Gee-Sern Jison Hsu, Marina Gavrilova

**Affiliations:** 1 Department of Computer Science, Faculty of Science, Main Campus, University of Calgary, Calgary, AB T2N 1N4, Canada; asmhossain.bari@ucalgary.ca (A.H.B.); mgavrilo@ucalgary.ca (M.G.); 2 Artificial Vision Laboratory, Department of Mechanical Engineering, National Taiwan University of Science and Technology, No. 1, Sec. 4, Roosevelt Rd., Taipei 10617, Taiwan; jison@mail.ntust.edu.tw

**Keywords:** remote visual technology, motion capture sensor, human motion, deep learning, long short-term memory, gait, emotion recognition, handcrafted features

## Abstract

Motion capture sensor-based gait emotion recognition is an emerging sub-domain of human emotion recognition. Its applications span a variety of fields including smart home design, border security, robotics, virtual reality, and gaming. In recent years, several deep learning-based approaches have been successful in solving the Gait Emotion Recognition (GER) problem. However, a vast majority of such methods rely on Deep Neural Networks (DNNs) with a significant number of model parameters, which lead to model overfitting as well as increased inference time. This paper contributes to the domain of knowledge by proposing a new lightweight bi-modular architecture with handcrafted features that is trained using a RMSprop optimizer and stratified data shuffling. The method is highly effective in correctly inferring human emotions from gait, achieving a micro-mean average precision of 0.97 on the Edinburgh Locomotive Mocap Dataset. It outperforms all recent deep-learning methods, while having the lowest inference time of 16.3 milliseconds per gait sample. This research study is beneficial to applications spanning various fields, such as emotionally aware assistive robotics, adaptive therapy and rehabilitation, and surveillance.

## 1. Introduction

Motion capture sensor-based gait emotion recognition from human motion is an emerging sub-domain of remote visual technologies. Remote visual technologies based on motion capture sensor and human motion have applications in a variety of domains, including smart home design, border security, unmanned aerial vehicles (UAVs), robotics, virtual reality, and gaming [1]. They have become even more essential in the current world of remote workplaces and virtual classrooms.

Recognizing emotions of the subject has fueled a number of human-related autonomous tasks [2]. Naturally, human emotion can be inferred through various biometric modalities that are affected by the subject’s emotions, such as facial expressions [3], gait [4,5,6], hand gestures [7], voice tones [8], and text [9]. The phenomenon of expressing emotions through human body movements has been observed from the early 1900s [10,11,12,13]. Consequently, Gait Emotion Recognition (GER) is defined as the process of determining emotions through a subject’s manner of walking [14]. GER can be beneficial in tuning and designing rehabilitation exercises [15], making emotionally intelligent robots [14,16], diagnosing and treating medical conditions [17,18], and identifying a person in distress during natural disasters [1]. The task requires body skeletal data (body joint coordinates over time) as the input, which can be collected from various types of motion capture sensors [19,20] or extracted from a video using pose-estimation methods [21,22,23]. These gait sequences can be processed to infer emotions from a set of discrete categories or mapped onto a continuous 3D space of features that can model various emotions [24]. Furthermore, the state of emotion of the subject can be collected from the participants themselves or from an audience observing their walking style. The latter is called perceived emotions.

Emotion recognition has been primarily researched using a person’s facial expressions, which has proven to be effective; however, gait-based emotion recognition systems have started gaining popularity recently [14]. Since gait can be collected in a non-intrusive manner, specifically remotely and without the subject’s cooperation, emotion recognition from gait has been adopted in smart home designs for fall prevention, disaster management during evacuation, and medicine for Parkinson’s detection and treatment [1]. Moreover, gait analysis can be performed with a low-resolution video or depth sensor data, where the face of the subject cannot be recognized [4].

There are two emotion representation models that are commonly accepted in research related to emotions [4]. They are the Distinct Categories model, which describes various emotions as mutually exclusive categories, and the Pleasure Dominance Arousal model, which treats emotions as a continuous spectrum. To ensure a clear interpretation and evaluation of the model, this article follows the Distinct Categories model with four emotion classes: angry, happy, sad, and neutral.

The recent works in the domain of GER have primarily focused on deep learning approaches to infer emotions using gait skeleton data either extracted from videos or collected directly using sensors. All the deep learning techniques for GER use Deep Neural Networks (DNN) with various layers, which result in an increased processing time and are detrimental to the model’s robustness and performance. This research paper addresses the following research questions:

1. Can a deep learning architecture for gait-based emotion recognition be designed to identify distinctive sequential and temporal features extracted from body joints?
2. Can a deep learning architecture combining sequential neural networks and multi-layered perceptrons be used to recognize emotions from human gaits?
3. Can the dynamic handcrafted features based on the geometric relationship between body joints be combined with the deep learning architecture to further improve recognition performance?
4. Can a light deep learning architecture be developed to ensure low inference time while ensuring high recognition performance?

Consecutively, the contributions of this paper are as follows. A novel hybrid deep learning architecture is presented that utilizes Long Short-term Memory (LSTM) units followed by Multi-layered Perceptrons (MLP) to extract a a distinctive feature map from raw gaits and recognize four emotions, namely happy, angry, sad, and neutral. Additionally, the proposed neural network has a significantly low number of parameters compared to prior research, which results in the lowest inference time among recent state-of-the-art deep learning-based GER methods. Furthermore, the proposed network takes advantage of geometric features, such as the Joint Relative Angle (JRA) and Joint Relative Distance (JRD), that are important for gait data processing. Finally, an extensive comparison of methods against the proposed hybrid deep learning architecture is performed on a benchmark dataset to demonstrate the superiority of the proposed method in terms of emotion recognition performance and inference time.

This research paper first briefly discusses the previous research performed in gait-based emotion recognition in Section 2, with a summary of the comparative deep learning-based methods presented in Table 1. Next, the paper describes the proposed method in Section 3, including a description of the dataset used to train and evaluate the proposed model. Then, the paper presents the experimentation results, showing that the proposed method has a higher recognition performance and a lower inference time than the state-of-the-art works in Section 4. Finally, the findings of this research study are summarized in Section 5 and the potential future directions for this domain are discussed.

## 2. Related Works

Emotion recognition systems can be implemented using different modalities. Speech-based emotion recognition systems rely on linguistic and tonal features present in voice recordings [25]. However, the extraction of such features is dependent on voice quality [25,26,27]. The dependence on clear recordings makes speech emotion recognition highly sensitive to noise. Additionally, certain voice characteristics might be interpreted as emotionally driven [25]. Alternatively, Facial Emotion Recognition (FER) systems depend on geometric and appearance features [28]. Although FER has proved to be a reliable method for emotion recognition, it is also dependent on high quality images or videos as its input. Moreover, the data required for FER may not be easily collectable for real world applications due to privacy concerns. In contrast, body movement data is considered to be less sensitive to noise and more acceptable in security applications [4], thus making collection easier. Body movements can be classified into sub-categories, namely gestures and gait, and either of them can be used for emotion recognition. However, research studies have shown that cultural background has a strong effect on human gestures [29,30]. Hence, the non-intrusive collection of non-sensitive gait data that is independent of cultural backgrounds is an appropriate choice to infer a subject’s emotions.

Gait is the representation of the behavioral patterns of a human via walking. Human activity can be recognized through hand gestures, expressions, behavior, etc. [31]. There are numerous strategies that have been developed for human activity recognition, such as (i) space–time, (ii) stochastic, (iii) rule-based, (iv) shape-based, (v) affective, (vi) behavioral, and (vii) social networking [31]. Additionally, due to the close relation of the two tasks, the intuitions behind a lot of techniques used for human activity recognition are relevant for GER. Most of the early works in GER were based mainly on machine learning algorithms, such as Principal Component Analysis (PCA) [32,33,34], and were aimed at producing information-rich features that eventually were used to infer the emotion expressed through the human gait. In 2010, Karg et al. [32] extracted three sets of features using velocity, stride length, and cadence, along with the minimum, mean, and maximum values of the joint angles. The authors then transformed these features using PCA, followed by a Fourier transform to obtain an information-rich feature set. The extracted feature set of this method was then used to classify the gaits into the emotion categories of happy, angry, sad, and neutral using various classifiers: the Support Vector Machine (SVM), Naïve Bayes (NB), and the Nearest Neighbor (NN). A similar approach was followed by Li et al. [34]. They used Discrete Fourier Transform (DFT) to obtain time and frequency-based features. Additionally, authors calculated features, such as step height, step length, step period, and walking velocity, and combined them with DFT features to form a set. This set was then dimensionally reduced using PCA and latent discriminant analysis, and used to infer an emotion. While discriminant analysis-based techniques were successful in showing that GER from skeleton gait data was possible, these methods were not accurate enough. Naturally, the research shifted towards discovering the features using more sophisticated methods.

In 2018, Ahmed et al. [5] introduced a methodology to extract a set of 17 features based on Laban Movement Analysis (LMA) from the input gait sequences. A subset of features was selected using a Genetic Algorithm (GA) for four different classifiers. Finally, the results of the classifiers were combined using score and rank-level fusion to improve the accuracy. This methodology was further improved upon by Ahmed et al. [6]. The authors identified ten groups of features crucial to GER. The feature set was then refined using Analysis of Variance (ANOVA) and Multivariate ANOVA (MANOVA) before undergoing another round of feature selection using a binary GA. Lastly, the final set of features were used to classify emotions using the score and rank-level fusion. Research using classical machine learning approaches for feature extraction and classification were robust and contributed to the domain by describing various groups of features that are beneficial for GER. However, methods relying only on handcrafted features had a limited set of features that was highly domain-specific. Furthermore, recent methodologies employing deep learning take advantage of data-driven approaches that rely on salient features and are applicable to much larger training datasets.

A popular deep learning approach for processing gait data involves the Graph-based Neural Network (GNN). In 2018, Yan et al. [35] devised a graphical representation of human skeleton data using graphs. Each joint of the skeleton from one frame was connected to the corresponding joint in the skeleton graph of another frame. The authors explored three types of partitioning for convolution operations on this graphical representation: uni-labelling, distance partitioning, and spatial configuration. This representation of skeleton data was strikingly close to an actual body’s skeleton; however, by ignoring joint distances and the direction of connections, it did not include the dependencies of bones and joints. Research by Bhattacharya et al. [36] made use of the Spatial Temporal Graph Convolutional Network (STGCN) from [35] to produce synthetic gaits and classify gaits into four emotion classes. They extended the STGCN architecture using a pooling layer and convolutional layer to create their model called STGCN for Emotion Perception (STEP).

Another widely adapted methodology for processing sequential gait data is the use of Recurrent Neural Networks (RNN) [37,38]. These works model the 2D or 3D gait sequences as a collection of body joints as a frame and the collection of the frames as the entire sequence. RNNs, such as Gated Recurrent Units (GRUs) and LSTM units, are used to process the sequential data and produce a feature set, which is in turn used to infer the output. Since RNNs explore relationships between features within a time-step and features across time-steps, these methods ensure that all possible dependencies are analyzed. Randhavane et al. [38] used an encoder–decoder style DNN to produce a synthetic set of gait sequences. The encoder from that network was then used as a feature extractor to produce deep features that were combined with posture-based and movement-based gait features. Finally, a random forest classifier produced an output in terms of four discrete emotions. A similar methodology was utilized in [37]. However, their approach failed to explore all possible dependencies within the body joints and relied on GRUs, which provided a limited control over the memory cells. Moreover, encoder–decoder models produce an embedding representative of the input gait but does not ensure that the embedding is optimal for the task of emotion recognition. A brief comparative summary of the deep learning-based methods is given in Table 1.

Our paper proposes, for the first time, an LSTM and MLP-based neural network with a sophisticated encoder-like design that learns the features hierarchically for GER. The LSTM sub-network in the proposed architecture facilitates the processing of sequential gait data by exploiting the spatial dependencies between all body joints in a frame, as well as by extracting temporal features using all the frames in a gait sequence. These extracted features are then consolidated and selected by the MLP sub-network. Hence, the two sub-networks work together to explore features using all possible combinations of body joints, followed by a selection which enables the architecture to discover latent features that might have been unaccounted for in the previous works. Moreover, this paper proposes a network specifically trained to identify emotions from recorded gait sequences. The proposed network hence exhibits an improved precision score when compared to the previous methods, while ensuring a light architecture that results in a low inference time. In addition, the network also utilizes geometric handcrafted features that amplify the features representing joint distances and the direction of connections to make the architecture robust to class imbalances in the dataset, which in turn results in a further increase in the performance.

**Table 1:** Summary of gait emotion recognition literature.

Article	Year	Methodology	Training Dataset	Pros	Cons
Karg et al. [32]	2010	PCA, Fourier transform, and dimension reduction on handcrafted features, followed by SVM, NB, and NN classifiers to classify	Dataset collected at Technische Universität München (TU München) [39]	Dimensionally reduced feature set.	Limited feature set consisting of only basic handcrafted features and classical machine learning algorithms. Low accuracy.
Yan et al. [35] (STGCN)	2018	Spatial Temporal Graph Convolutional Network (STGCN)	DeepMind Kinetics video dataset [40]	Structured graph representation of gait skeleton and three different partitionings for graph convolutions.	Dependencies between bones and joints not exploited.
Ahmed et al. [6]	2019	ANOVA and MANOVA for feature refinement, and GA for feature selection, followed by a score and rank-level fusion of four classifiers	Proprietary dataset [5]	Two-layered feature selection from 10 pools of features.	Classical machine learning algorithms.
Bhattacharya et al. [36] (STEP)	2020	Concatenation of affective features and features extracted from STGCN	E-Gait dataset [36]	CNN for processing STGCN output and hybrid feature set.	Dependencies between bones and joints not exploited.
Randhavane et al. [38] (ADF)	2020	Concatenation of affective and LSTM-extracted deep features	EWalk dataset [38]	Hybrid feature set and a dedicated classifier.	Inefficient LSTM module.
Bhattacharya et al. [37] (HAPAM)	2020	Hierarchical attention pooling and affective mapping using GRUs	Emotion–gait dataset [37]	Hierarchical network and hybrid features.	GRUs used instead of LSTM units.

## 3. Methodology

### 3.1. Proposed LSTM and MLP Sub-Networks

As discussed in Section 2, deep learning methodologies for identifying emotions through gait patterns showed a great potential in comparison to conventional machine learning methods. This is due to the fact that neural networks are data-driven and can discern latent features that might be overlooked by conventional machine learning methods.

Since gait is a time-series data containing coordinates for each joint in the subject’s gait skeleton, it is imperative to employ sequential data processing methods to extract temporal features. Furthermore, exploration of the spatial features exhibited by the gait skeleton in each frame is also important for processing gait data. Hence, the first half of the proposed deep learning architecture is made up of neural networks that are proficient at processing sequential data to extract spatial as well as temporal features. Although (Graph Convolutional Networks) GCNs can be used to produce features from both dimensions (time and space), LSTMs have certain advantages over GCNs when it comes to processing gait data. Graph convolutions work by aggregating features from neighboring nodes and using that to update the current node’s features. In a typical representation of the human body, these nodes represent the various body joints. Consequently, when low-level features in the initial stages of graph convolutions are being computed, only the features from neighboring joints are combined. As a result, low-level features in a given frame that are dependent on body joints/graph nodes far away from one another are unaccounted for. Similarly, the low-level features dependent on body joints that are in far apart time frames of a gait sequence are also ignored. To address the issues mentioned above, the architecture proposed in the paper utilizes LSTM units in the first half of the network. These units explore low-level features in the temporal as well as the spatial dimension by employing memory cells to calculate features across frames and a weight matrix to extract features within a frame, respectively.

Furthermore, despite GRUs and LSTMs both being popular choices for long sequences, the proposed network is built using LSTM layers due to the reasons discussed below. The LSTM can be described by the following equations:

(1) $$i_{t}=Tanh\left(W_{ix}x_{t}+W_{ia}a_{t-1}\right)$$

(2) $$f_{t}=\sigma \left(W_{fx}x_{t}+W_{fa}a_{t-1}\right)$$

(3) $$o_{t}=\sigma \left(W_{ox}x_{t}+W_{oa}a_{t-1}\right)$$

(4) $$c_{t}=f_{t}\cdot c_{t-1}+i_{t}\cdot Tanh\left(W_{ox}x_{t}+W_{oa}a_{t-1}\right)$$

(5) $$a_{t}=o_{t}\cdot c_{t}$$

In the above equations, $W_{mn}$ represents the weight matrix for gate *m* and input *n*, σ represents the sigmoid activation function, and Tanh represents the hyperbolic tangent activation function. A typical LSTM unit consists of three gates, namely input, forget, and output gates, which govern the decision of storing a value in the memory cell of the LSTM. As shown in Equations (1)–(3), the gates depend on the current input $\left(x_{t}\right)$ and the previous activation (at−1). As a result, the forget gate $\left(f_{t}\right)$ and input gate $\left(i_{t}\right)$ values regulate the effect of both the previously stored memory (ct−1) and the current output, as shown in Equation (4). Hence, during the calculation of the new memory value to be stored, namely $\left(c_{t}\right)$, there are two different gates (forget and input) at play. Finally, the activation for the current time step $\left(a_{t}\right)$ is computed as the dot product between the output gate $\left(o_{t}\right)$ and the memory cell $\left(c_{t}\right)$, as shown in Equation (5). In contrast, GRUs rely on only the input gate to control the effect of both the previous activation and the stored memory (Equation (6) describes the update of the memory cell in a GRU). Hence, while LSTMs have slightly higher number of parameters, they offer more control over the updates of the memory cell and eventually result in better learning for sequences.

(6) $$c_{t}=\left(1-i_{t}\right)\cdot c_{t-1}+i_{t}\cdot a_{t-1}$$

Once the LSTM sub-network produces the feature set, the features must be consolidated to produce the classification results. While using a conventional machine learning classifier on extracted latent features can yield good results, a neural network-based classifier performs better with sufficient amounts of data. Furthermore, due to their ability to automatically extract and select new discriminating features, MLPs are a great choice for the selection of latent features and for subsequent classification. This ability of MLP, paired with the tapered design of the second sub-network, allows the proposed model to produce a feature set that is smaller but contains more information-rich features. Therefore, the functionality of the MLP sub-network is twofold: feature extraction to produce a condensed feature set and mapping those features to the various emotion classes.

### 3.2. Overall Deep Learning Architecture

**Figure 1:**
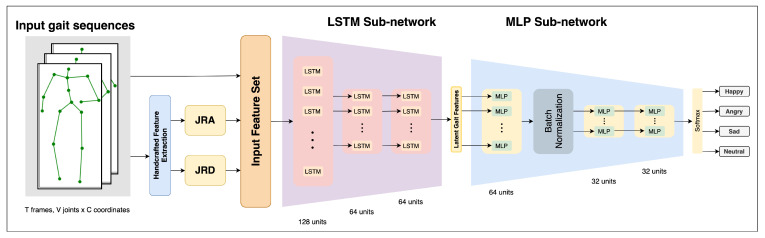
Architecture of the proposed bi-modular sequential neural network.

The proposed deep learning architecture is comprised of two modules: the LSTM sub-network and the MLP sub-network, as shown in Figure 1. The overall architecture of the network has a tapered designed to produce a condensed representation of the input by ensuring that the amount of information received at the input is represented by a smaller set of features towards the end. This is achieved through an architecture where the next layer has either an equal or smaller number of units than the previous one. Such a design also ensures that the network has a low number of parameters since the number of units in each layer decreases with the depth of the network. The network is trained with a categorical cross entropy loss for each of the emotion classes, which ensures that network weights are trained specifically to extract features relevant for the task of emotion recognition.

The network accepts an input vector of size [T,(V*C)+F], where *T* is the number of time steps in each gait sequence, *F* is the size of the handcrafted feature set, *V* is the number of body joints in the body skeleton, and *C* is the number coordinates for each body joint (thus, for an input of only raw gait sequences, F=0). This sequential gait input is processed by a three-layered LSTM sub-network containing 128, 64, and 64 LSTM units. All of these layers have a hyperbolic tangent (Tanh) activation function to ensure that the negative activation values are not ignored while introducing non-linearity. The resulting feature set of size 64 is passed onto the MLP sub-network, which has three layers with decreasing number of units (64, 32, and 32) with Tanh activations. Additionally, the activations of the first MLP layer are batch-normalized with a momentum of 0.1 to promote regularization. This sub-network is responsible for producing a denser feature set of 32 features that is mapped to the four emotion classes, after which a Softmax activation is used to convert the scores into probabilities. Lastly, the class with the highest probability value is chosen.

### 3.3. Handcrafted Features

While using a purely deep learning approach has its advantages, it can be further empowered by the previous knowledge from the problem domain. The data-driven approach of deep learning methodologies is highly influenced by the dataset used to train the deep learning network. This characteristic can cause poor performance for classes in the dataset which do not have as many number of samples as the other classes. The problem can be mitigated by avoiding relying solely on deep learning. Therefore, the proposed architecture incorporates robust handcrafted gait features as well. One way to make use of the sequential handcrafted features with a deep learning architecture is to concatenate them with the output of the LSTM sub-network before feeding that to the MLP sub-network. However, the features must be non-sequential to be processed directly by the MLP sub-network. This can be done by utilizing statistical values that hold information about the sequential data; for instance, this includes the mean or the highest value of the series. Unfortunately, such values ignore the latent features present in that sequential data that cannot be computed using statistics. Another way is to incorporate the handcrafted features at the score level. By simply training a separate classifier on handcrafted features, in addition to the deep learning model, one can combine the scores of each class from the two models. However, this means that the decisions made on the different types of feature sets are considered, eliminating the possibility of making a decision using both of the information sources. Therefore, the network is designed to accept a feature set as its input, which contains two handcrafted features in addition to raw gait sequences. These features, along with the raw gait sequences, are used to produced higher-level features in the first half of the network, which are then refined and classified into emotions by the second half of the network.

To find the most suitable feature set for the model, combinations of four handcrafted features that are important for processing gait data are considered [41,42]. The four features, namely Joint Relative Angles (JRAs), Joint Relative Distances (JRDs), Joint Relative Triangle Areas (JRTAs), and Joint Relative Cosine Dissimilarities (JRCDs), can describe the geometric and directional motions of a subject’s body joints [42], consequently improving the performance of the network. All the handcrafted features mentioned below are computed for each frame in a gait sequence.

The JRA between two body joints, namely $A(x_{1},y_{1},z_{1})$ and $B(x_{2},y_{2},z_{2})$, is the angle formed at the mid-spine joint $S(x_{0},y_{0},z_{0})$ between the vectors $\vec{SA}$ (vector from the mid-spine joint to body joint *A*) and $\vec{SB}$ (vector from the mid-spine joint to body joint *B*). The angle is defined as the inverseCosine of the dot product of $\vec{SA}$ and $\vec{SB}$ over the product of the magnitudes of the two vectors. Equation (7) describes the calculation mathematically. The V−1 body joints (excluding the mid-spine joint) result in V−1 vectors originating from the mid-spine joint, which are used to calculate the $\left(\frac{V-1}{2}\right)$ angles formed between all possible pairs of vectors. Thus, the size of the JRA feature set is (T,(V−12)), where *T* is the number of time steps in the gait sequence. This feature is representative of the relative angular motions of the various body joints. Furthermore, a stable joint that remains mostly stationary throughout the gait should be chosen as the relative joint. Hence, the mid-spine is chosen as the relative joint for all the features [42].

(7) $$\theta =Cos^{-1}\left(\frac{\vec{SA}\cdot \vec{SB}}{\left|\vec{SA}\right|\;\left|\vec{SB}\right|}\right)$$

Additionally, the JRD considers the relative motion of various body joints in terms of distance [41]. The JRD between two points, namely $A(x_{1},y_{1},z_{1})$ and $B(x_{2},y_{2},z_{2})$, is calculated as the Euclidean distance between the two joints. The mathematical formula for this calculation is mentioned in Equation (8). Although Euclidean distance is a commonly used metric for gait analysis tasks, other distance metrics, such as Mahalanobis and city block, can be potentially used for calculating JRDs. However, there is no evidence in gait recognition literature suggesting that other metrics are more beneficial than the Euclidean metric [1]. Similar to JRAs, V2 JRDs are calculated for all possible combinations of two joints in the body skeleton for one frame of the gait sequence.

(8) $$Euclidean\;Distance\left(A,B\right)=\sqrt{{\left(x_{2}-x_{1}\right)}^{2}+{\left(y_{2}-y_{1}\right)}^{2}+{\left(z_{2}-z_{1}\right)}^{2}}$$

Two more features, namely JRTA and JRCD, as introduced in [42], were considered as a part of the input feature set. However, when comparing the feature set of raw gait sequences with JRA and JRD (introduced in [41]) as well as with JRTA and JRCD, it was found that JRTA and JRCD do not increment the model precision. Hence, the final feature set included raw gait sequences, JRAs and JRDs, of size [T,(V*C)+(V−12)+(V2)], where *T* is the number of time steps in a gait sequence, *V* is the number of body joints for each time step, *C* is the number of coordinates for each joint in a time step, and (V−12) and (V2) are the sizes of the handcrafted feature set of JRA and JRD, respectively.

In addition to choosing the most appropriate input feature set for the network, using optimal hyper-parameters is essential for a deep learning model to train well on the data and eventually exhibit a high performance. Hence, the model was experimented on using different hyper-parameter values that will be discussed in the next section.

## 4. Experimental Results

**Figure 2:**
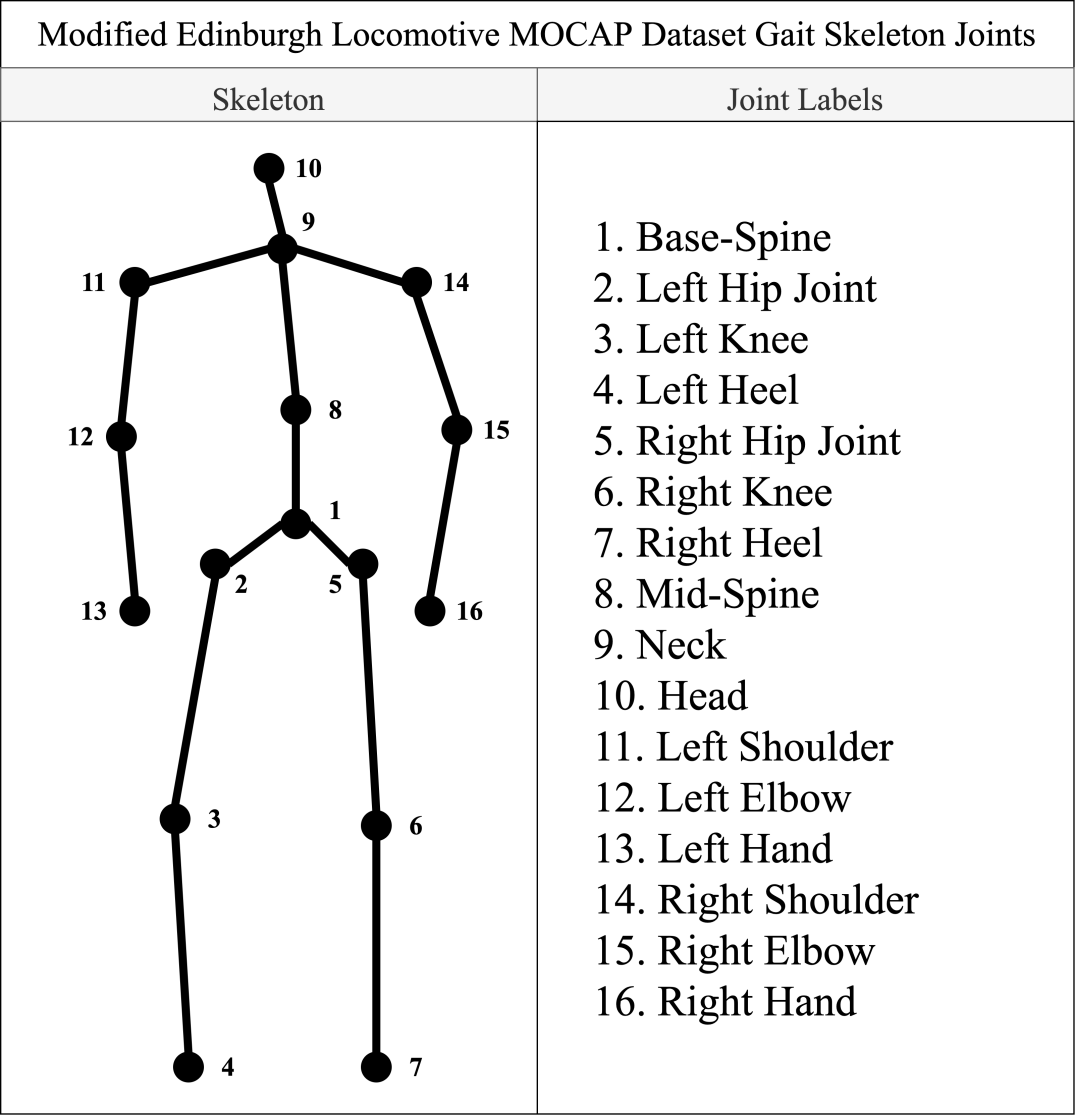
Modified body skeleton from Edinburgh Locomotive MOCAP Dataset.

This section describes the numerous experiments that were run to tune the hyperparameters of the network as well as to train and test the proposed network. All the experiments discussed in this paper were run with a data split of 80:10:10 for training, validation, and testing using a stratified shuffling on the Edinburgh Locomotion Mocap Dataset (ELMD), which was collected by researchers from the University of Edinburgh [43] and annotated by Bhattacharya et al. [37]. The modified ELMD dataset consists of 1835 gait sequences recorded for 4 s at 60 Hz. Thus, each gait sequence in the dataset has 240 frames. Each gait sequence consists of 48 values which correspond to three coordinates of 16 body joints (see Figure 2 for details). While GER can be performed using gait skeletons with a fewer number of body joints and/or lower dimensional coordinates, having fewer amounts of data points would be detrimental to the network’s performance. These gait sequences were labeled by [37] into four categories of emotions: angry, happy, sad, and neutral. The labels were generated by various participants on a crowd-sourced website that provided ratings to each sequence based on the emotions they perceived from that gait. This resulted in a dataset of 1835 gait sequences, with each sequence consisting of 240 frames and each frame with 16 × 3 values. These 1835 gait sequences consist of 1048 angry gaits, 454 happy gaits, 254 sad gaits, and 79 neutral gaits.

The ELMD dataset is the benchmark dataset for emotion recognition research from gait. This dataset is very popular since it is emotionally labeled gait data, which is not artificially synthesized and has a sufficient number of samples for deep learning training. It has, however, an unequal number of class samples corresponding to different emotions. In all of the prior research, higher precision values were observed for the classes with more samples and vice versa. On a positive note, this attribute of the dataset also allows for the identification of methods that are too dependent on the class sample distribution.

### 4.1. Evaluation Metrics

The performance of the proposed bi-modular sequential network was evaluated using mean Average Precision (mAP) metrics. The macro mAP was computed as the mean of all the class average precision scores (described in Equation (9), where $AP_{i}$ is the average precision for class *i* and *N* is the total number of classes). As opposed to macro-mAP, micro-mAP considers each sample as a unique class. This metric is suitable for unbalanced datasets such as the one used in this research study since it considers the varied representation of classes in the dataset. The micro-mAP measure is defined mathematically in Equation (10), where $TP_{i}$ and $FP_{i}$ are the number of true positives and false positives for class *i*, respectively, and *N* is the number of classes (which is equal to the number of samples for micro-mAP). Hence, micro-mAP measures the number of samples classified correctly irrespective of their classes and macro-mAP represents the model’s overall performance with respect to all the classes.

(9) $$macro\;mean\;Average\;Precision=\frac{AP_{1}+AP_{2}+\ldots +AP_{N}}{N}$$

(10) $$micro\;mean\;Average\;Precision=\frac{TP_{1}+TP_{2}+\ldots +TP_{N}}{TP_{1}+TP_{2}+\ldots +TP_{N}+FP_{1}+FP_{2}+\ldots +FP_{N}}$$

### 4.2. Optimizer Selection Experiment

**Figure 3:**
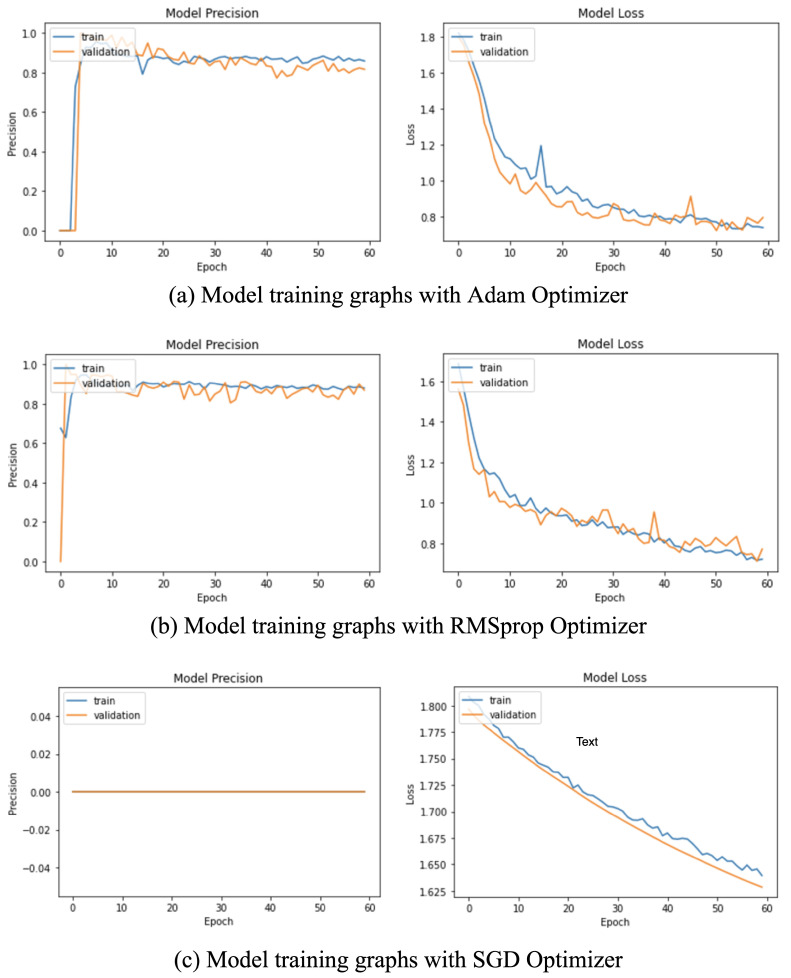
Model training graphs with: (**a**) Adam optimizer, (**b**) RMSprop optimizer, and (**c**) SGD optimizer.

The proposed LSTM and MLP networks were trained using the Adam, RMSprop, and SGD optimizers. The Adam and RMSprop optimizers led to steep training loss graphs which showed a decrease in the network loss. In contrast, the SGD optimizer provided a smooth learning curve for the network but was unable to achieve a good performance; see Figure 3. Clearly, RMSprop can be identified to have the best overall performance, followed by the Adam optimizer (see Table 2). While the Stochastic Gradient Descent (SGD) provided a smooth learning for the network, it failed to produce optimal results. Hence, the RMSprop optimizer was selected as the optimizer for the model.

### 4.3. Activation Function Selection Experiment

**Figure 4:**
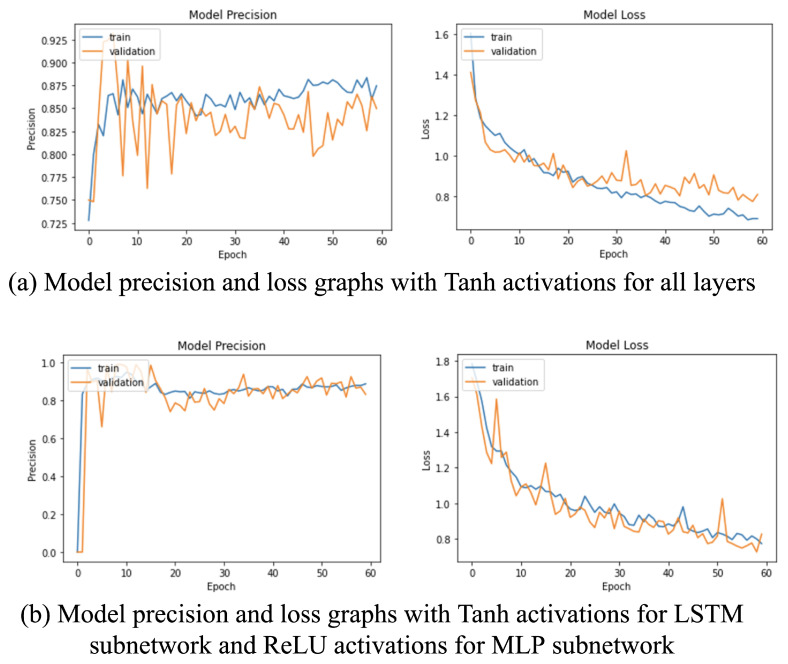
Model training graphs with: (**a**) TanH activation functions in all layers of the network and (**b**) TanH activations in the LSTM sub-network and ReLU activation functions in the MLP sub-network.

Next, the network was tested using two different activations: Tanh and the rectified linear unit (ReLU). While using ReLU activations in MLP resulted in a faster learning and closer loss curve (see Figure 4), the model could not achieve a high mean average precision (mAP) value on the test set. However, the weights could reach their optimal values using Tanh activations in all layers, which is evident from the higher mAP value observed in Table 3. Moreover, using ReLU activations for the proposed LSTM sub-network resulted in the dying ReLU problem, which occurs when a large number of neurons are zeroed out.

### 4.4. Dropout Selection Experiment

The network was also tested using different dropout values and positions, and the configuration that yielded the best results was a dropout in the second layer of the MLP sub-network with a dropout value of 0.2, which resulted in a network that had a mean average precision of 0.955 on the test set, as seen in Table 4. A dropout of 0.1 in the first layer of the MLP sub-network also increased the mean average precision score; however, the regularization was less apparent. Hence, a dropout of 0.2 in the second layer of the MLP sub-network was chosen.

### 4.5. Data Shuffling Selection Experiment

**Figure 5:**
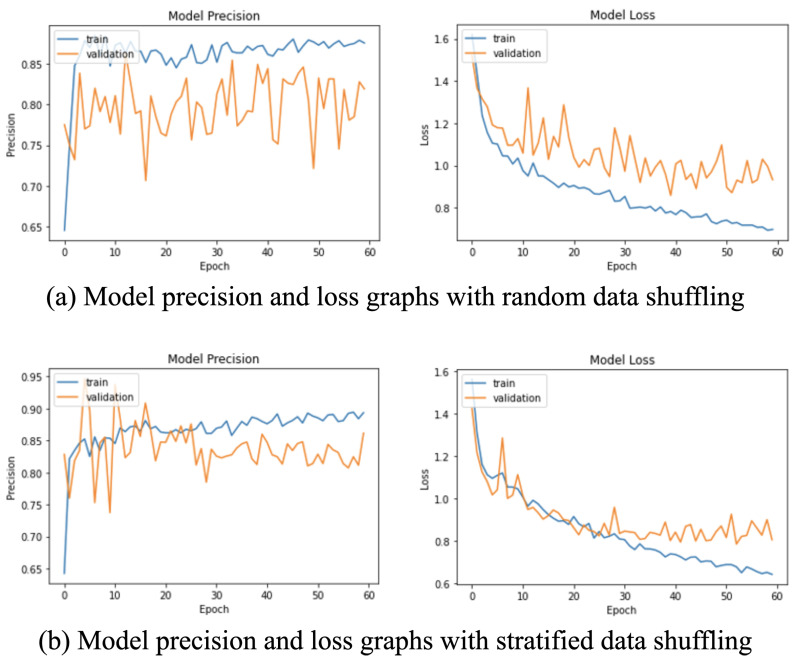
Model training graphs with (**a**) random and (**b**) stratified data shuffling. The stratified data shuffling results in less overfitting and a lower model loss. Hence, stratified data shuffling was used.

The network was trained using a typical 80:10:10 data split of the ELMD dataset [43] with two types of data shuffling: random and stratified. Using stratified data shuffling resulted in more generalized training, which can be observed as close trends of the training, validation loss, and precision curves in Figure 5. Consequently, this improved the precision score of the model, as shown in Table 5, and hence stratified shuffling was chosen for this model.

### 4.6. Batch Size Selection Experiment

**Figure 6:**
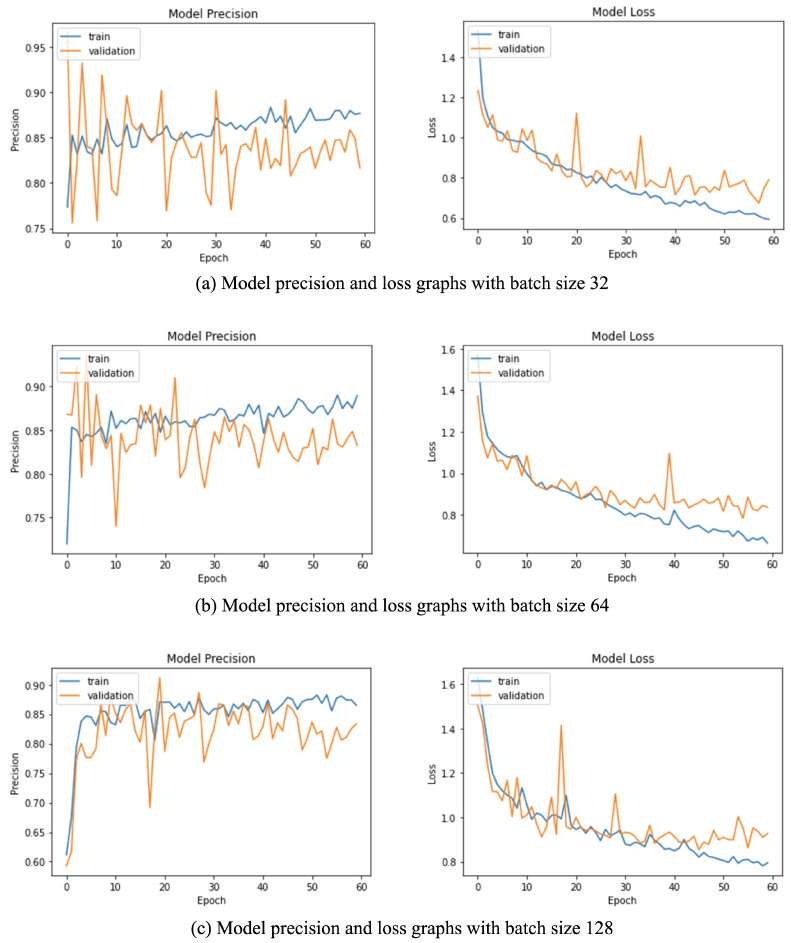
Model training graphs with smaller batch sizes result in unstable learning depicted by the oscillating precision and loss curves. In contrast, larger batch sizes ensure that batches have a good representation of input samples from each class. However, larger batch sizes also cause the learning to regularize too much and worsens the performance of the network. A batch size of 64 provides a balance of smooth learning with a low loss value at the end of the training and was chosen as a model parameter.

Various batch sizes were used with the network to ensure that a sufficient number of examples are processed by the network before updating its weights. Table 6 displays the various batch sizes that were used to train the network and how the precision scores changed with this parameter. The respective precision and loss curves can be seen in Figure 6. Smaller batch sizes resulted in more unstable loss curves due to the insufficient number of samples from all classes in the batch, while bigger batch sizes resulted in reduced variations during training and hence smoother loss curves. Consecutively, a batch size of 64 ensured that each class sample had appropriate representation in a batch and hence resulted in the highest mean average precision values while ensuring that the model did not overfit the data. This behavior can be observed as close training and validation loss curves in Figure 6b and the high mAP value for a batch size of 64 in Table 6.

### 4.7. Number of Epochs Selection Experiment

**Figure 7:**
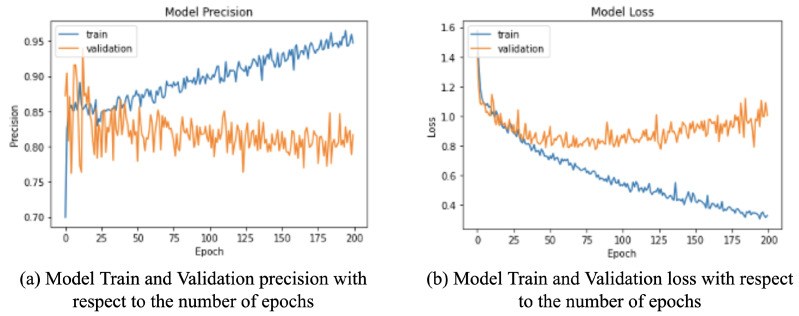
Model precision and loss graphs for the (**a**) training and (**b**) validation sets with respect to the number of epochs. The validation loss of the model started to stabilize around epoch 75 and then began to overfit. Additionally, the precision values of the model also started dipping shortly after epoch 75. Hence, the number of epochs to train the model was chosen to 75.

The network performance was also recorded with respect to the number of epochs to find the optimal number of epochs where the network performance peaks before it starts overfitting. As shown in Figure 7, the model’s validation loss consistently decreased until epoch 75, after which it started increasing, signifying overfitting beyond that point. Hence, the number of epochs to train the network was chosen to be 75.

### 4.8. Learning Rate Selection Experiment

While tuning the network, different learning rates were also considered in order to find the optimal configuration. The learning rate for the training of the network was optimized by testing out various rates in an incremental fashion. The model exhibited the highest performance for the learning rate of 1 × 10^−4^.

### 4.9. Optimal Network Configuration

**Figure 8:**
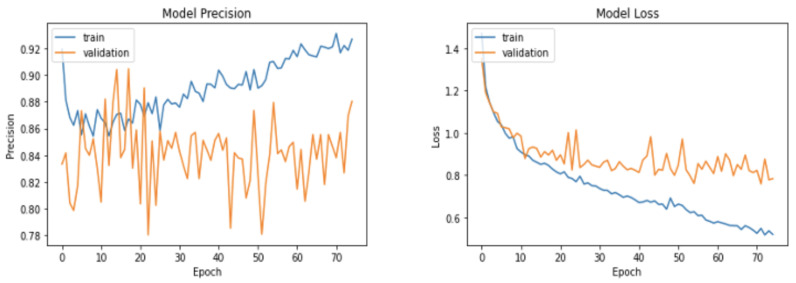
High regularization in the model training graphs due to the batch normalization after the first MLP layer.

Based on the experimental results discussed in Table 2, Table 3, Table 4, Table 5, Table 6, Table 7 and Table 8, the configuration for the proposed LSTM and MLP network was finalized. The resulting network, as shown in Figure 1, is comprised of two main modules, namely the LSTM sub-network (128, 64, and 64) and MLP sub-network (64, 32, and 32), and all the network layers, except the output layer, use a Tanh activation function. Moreover, the number of parameters of this network while processing raw gait sequences, in addition to JRAs and JRDs, are only 295,684 as opposed to the 40 million parameters in other methods (see Table 8). The network was trained using RMSprop with *m* = 0.5 (momentum), ρ = 0.3 (rho), ϵ = 1 × 10^−7^ (epsilon), and a learning rate of 1 × 10^−4^, as mentioned in Section 4, with a batch size of 64 for 75 epochs. In order to further improve the system’s performance, different normalization methods were considered after each of the MLP and LSTM layers. The model showed an improvement in the overall performance and a significant increment in the regularization (see Figure 8) when batch normalization with a momentum of 0.1 after the first MLP layer was used. The data used was split as 80:10:10 for creating the training, validation, and test set, and was stratified.

### 4.10. Performance Comparison Experiments

The proposed bi-modular architecture was compared with the recent best performing state-of-the-art methods: STGCN (Spatial Temporal Graph Convolutional Network) [35], ADF (Affective and Deep Features) [38], STEP (CNN-based hybrid model) [36], and HAPAM (Hierarchical Attention Pooling and Affective Mapping) [37]. All of the methods were re-implemented using PyTorch; trained using the emotion–gait dataset [37] using their respective optimal training parameters with a 80:10:10 data split; and tested on the Edinburgh Locomotive Mocap Dataset, with the emotion class annotations provided by [37]. As seen from Table 7, the proposed bi-modular sequential neural network with JRA and JRD-handcrafted features as well as batch normalization outperformed all of the recent gait emotion recognition deep learning-based methods by attaining an overall micro-mAP value of 0.97 and macro-mAP value of 0.86. The micro-mAP was higher by 0.09 than that of the best performing method HAPAM [37], higher by 0.5 than that of method [35], and higher by 0.7 than [36,38]. The same architecture also achieves the highest macro-mean average precision value of 0.86, outperforming the best method of HAPAM [37] by 0.2.

The proposed model demonstrated a superior performance on all individual emotions as well, with the class AP score of over 0.9 for the angry, happy, and sad classes. It is worth noting that adding batch normalization also resulted in the increase of the neutral class emotion recognition from 0.46 in the proposed architecture, with handcrafted features set to 0.65. Due to the lack of samples for this emotional class, it has proven to be the most difficult to recognize for all models, with the best prior result of only 0.18 reported in HAPAM [37]. The average precision of the proposed architecture, namely 0.65, for the neutral class is three times-improved over the best prior method value of 0.18.

The previous best method [37] is highly affected by the imbalanced class distribution of the dataset, which is observed as the highest performance for the angry class, followed by the happy, sad, and neutral classes. This is due to the fact that it is a purely data-driven approach and is hence more sensitive to the class variations in the data. However, this behavior is subdued in the proposed bi-modular neural network with the employment of powerful handcrafted features and batch normalization for network regularization. Furthermore, methodologies using graph NNs and combinations of both handcrafted and deep features tend to be biased towards the happy class. As a result, more gaits are wrongly classified by [35,36,38] as happy ones, which explains the low class and mean average precision scores. It is clear from the above discussion that the proposed LSTM and MLP architecture with handcrafted features and batch normalization outperforms all the above methods by leveraging (1) powerful sequential neural networks to extract temporal and spatial features, (2) tapered neural network architecture to consolidate those features, (3) handcrafted features to reduce the sensitivity towards class imbalances in the dataset, and (4) a light architecture to infer emotions faster.

The last metric that was used to compare the performances of the various GER methods was their respective inference times for one gait sample. The implementation of the proposed bi-modular neural network was done using Keras, while the implementation for all the other methods was done using PyTorch. These experiments were run on the standard version of Google Colaboratory Notebook, which uses a Tesla K80 GPU with 12 GB of GDDR5 VRAM, a dual-core Intel Xeon @ 2 GHz CPU, and a memory size of 13.3 GB. Each method was tested on the testing set derived from the ELMD dataset [43]. All of the three proposed bi-modular neural networks were observed to have the fastest inferences among all the comparators of less than 17 milliseconds, with the architecture based on only raw gait sequences being the fastest. This can be accredited to the fact that it has significantly fewer parameters in comparison to the other methods, as seen in Table 8.

## 5. Conclusions and Future Work

A novel LSTM and MLP architecture for gait emotion recognition has been proposed in this paper. The proposed bi-modular neural network architecture has only a fraction of the number of parameters compared to the current state-of-the-art methods, which allows for a fast inference of gait samples while outperforming the other methods on the ELMD dataset. The architecture relies on raw gait sequences as well as geometric handcrafted features that allow it to explore salient features in the gait sequence, in addition to being tolerant to class imbalances in the dataset. Furthermore, the LSTM units in the network allow it to extract low-level features from far away body joint as well as from joints that are further from each other in the temporal domain. Lastly, an extensive experimentation was performed to ensure that the network uses optimal hyperparameters while inferring emotions from gait sequences, which resulted in the highest micro-mean average precision of 0.97 compared to all other state-of-the-art methods re-implemented on the benchmark dataset. The ability to identify human emotion from gait is highly applicable to a variety of fields including robotics, affective computing, therapy, rehabilitation, and surveillance.

An interesting approach for future investigation involves hierarchical deep learning models which process joint trajectories independently and eventually combine the learned features from those trajectories to identify emotions. Additionally, the accurate representation of the human body has increased GER performance; hence, exploring deep learning architectures capable of processing directed body skeleton graphs is a promising avenue. Lastly, architectures employing 3D convolutional operations on gaits embedded as images can also be investigated for the extraction of spatial and temporal features for gait emotion recognition.

## Figures and Tables

**Table 2:** Performance of the proposed bi-modular sequential neural network with different optimization methods.

Optimizer Selection Experiment					
***Optimizer***	***Class AP (angry)***	***Class AP (happy)***	***Class AP (sad)***	***Class AP (neutral)***	***Micro-mAP***
Adam	0.991	0.802	0.694	0.360	0.915
**RMSprop**	**0.995**	**0.904**	**0.835**	**0.460**	**0.955**
SGD	0.876	0.436	0.217	0.265	0.679

RMSprop achieves the best mean and class average precision scores.

**Table 3:** Performance of the proposed method with different configurations of Tanh and ReLU activations.

Activation Function Selection Experiment					
***Activation Function Configuration***	***Class AP (angry)***	***Class AP (happy)***	***Class AP (sad)***	***Class AP (neutral)***	***Micro-mAP***
**Tanh for all layers.**	**0.995**	**0.904**	**0.835**	**0.460**	**0.955**
Tanh in LSTM sub-net and ReLU in MLP sub-net.	0.987	0.698	0.665	0.283	0.904

The configuration with TanH activations in all layers of the network results in the best mean and class average precision scores.

**Table 4:** Performance evaluation of proposed method with different values and positions of the dropout layer.

Dropout Position and Value Selection Experiment						
***Dropout Position***	***Dropout Value***	***Class AP (angry)***	***Class AP (happy)***	***Class AP (sad)***	***Class AP (neutral)***	***Micro-mAP***
No Dropout	-	0.990	0.827	0.542	0.509	0.920
1st	0.1	0.991	0.841	0.659	0.462	0.923
LSTM	0.2	0.991	0.746	0.490	0.436	0.902
Layer	0.4	0.990	0.853	0.765	0.304	0.928
2nd	0.1	0.988	0.780	0.547	0.350	0.906
LSTM	0.2	0.994	0.880	0.739	0.488	0.925
Layer	0.4	0.985	0.795	0.699	0.363	0.906
3rd	0.1	0.986	0.834	0.430	0.465	0.896
LSTM	0.2	0.994	0.764	0.398	0.314	0.906
Layer	0.4	0.978	0.762	0.505	0.504	0.877
1st	0.1	0.994	0.828	0.636	0.495	0.931
MLP	0.2	0.990	0.813	0.597	0.514	0.923
Layer	0.4	0.985	0.732	0.544	0.374	0.903
**2nd**	0.1	0.984	0.737	0.450	0.370	0.896
**MLP**	**0.2**	**0.995**	**0.904**	**0.835**	**0.460**	**0.955**
**Layer**	0.4	0.988	0.739	0.599	0.380	0.912
3rd	0.1	0.984	0.840	0.661	0.367	0.923
MLP	0.2	0.980	0.733	0.536	0.241	0.895
Layer	0.4	0.985	0.757	0.651	0.319	0.897

A dropout of 0.2 in the second layer of the MLP sub-network results in the best mean and class average precision scores.

**Table 5:** Comparison between random and stratified data selection methods for LSTM and MLP with JRA and JRD.

Data Shuffling Selection Experiment					
***Data Shuffling Method***	***Class AP (angry)***	***Class AP (happy)***	***Class AP (sad)***	***Class AP (neutral)***	***Micro-mAP***
Random	0.992	0.865	0.690	0.389	0.940
**Stratified**	**0.995**	**0.904**	**0.835**	**0.460**	**0.955**

Stratified data shuffling results in the best mean and class average precision scores.

**Table 6:** Performance of the proposed architecture with different batch sizes.

Batch Size Selection Experiment					
***Batch Size***	***Class AP (angry)***	***Class AP (happy)***	***Class AP (sad)***	***Class AP (neutral)***	***Micro-mAP***
16	0.987	0.863	0.776	0.544	0.938
32	0.993	0.815	0.785	0.487	0.928
**64**	**0.995**	**0.904**	**0.835**	**0.460**	**0.955**
128	0.988	0.829	0.677	0.400	0.913
256	0.986	0.810	0.589	0.417	0.915

A batch size of 64 achieves the optimal mean and class average precision scores.

**Table 7:** Performance comparison of the recent methods with the proposed bi-modular sequential neural network.

Performance Comparison Experiments						
***Method***	***Class AP (angry)***	***Class AP (happy)***	***Class AP (sad)***	***Class AP (neutral)***	***Macro-mAP***	***Micro-mAP***
STEP (2020) [36]	0.22	0.52	0.30	0.12	0.29	0.27
ADF (2019) [38]	0.22	0.59	0.30	0.12	0.31	0.27
STGCN (2018) [35]	0.06	0.97	0.20	0.01	0.34	0.41
HAPAM (2020) [37]	0.97	0.66	0.40	0.18	0.60	0.88
**Proposed LSTM** **and MLP (RGS)**	**0.98**	**0.74**	**0.58**	**0.33**	**0.66**	**0.92**
**Proposed LSTM** **and MLP (RGS + JRA + JRD)**	**0.99**	**0.90**	**0.84**	**0.46**	**0.80**	**0.96**
**Proposed LSTM and MLP with ** **batch normalization** **(RGS + JRA + JRD)**	**0.99**	**0.91**	**0.90**	**0.65**	**0.86**	**0.97**

The proposed bi-modular networks outperform the previous state-of-the-art methods in mean average precision scores.

**Table 8:** Comparison of the inference time of the recent methods with the proposed bi-modular sequential neural network.

Inference Time Comparisons		
***Method***	***Number of Parameters***	***Inference Time (in Seconds)***
STEP [36]	717,987	4.82 × 10^−2^
HAPAM [37]	40,444,854	4.66 × 10^−2^
ADF [38]	310,978	3.91 × 10^−2^
STGCN [35]	2,628,290	2.17 × 10^−2^
Proposed LSTM and with batch normalization (RGS + JRA + JRD)	**295,940**	**1.63 × 10^−2^**
**Proposed LSTM and MLP (RGS + JRA + JRD)**	**295,684**	**1.62 × 10^−2^**
**Proposed LSTM and MLP (RGS)**	**180,484**	**8.83 × 10^−3^**

The proposed bi-modular networks exhibit the fastest inference times.
